# Comparative Analysis of Indices for Social Determinants of Health in Pediatric Surgical Populations

**DOI:** 10.1001/jamanetworkopen.2024.49672

**Published:** 2024-12-10

**Authors:** Caroline Q. Stephens, Ava Yap, Lan Vu, Jacqueline M. Saito, Dwight Barry, Amy M. Shui, Hannah Cockrell, Sarah Cairo, Derek Wakeman, Loren Berman, Sarah Greenberg, Allison F. Linden, Jonathan Kohler, KuoJen Tsao, Nicole A. Wilson

**Affiliations:** 1 University of California, San Francisco, San Francisco; 2 Children’s National Hospital, Washington, District of Columbia; 3 Seattle Children’s Hospital, Seattle, Washington; 4 University of Rochester Medical Center, Rochester, New York; 5 Nemour’s Children’s Hospital, Wilmington, Delaware; 6 Emory University, Children’s Healthcare of Atlanta, Atlanta, Georgia; 7 University of California, Davis, Sacramento; 8 University of Texas Health Science Center, Houston, Texas

## Abstract

**Importance:**

Conclusions vary substantially among studies examining associations between area-based social determinants of health (SDOH) and pediatric health disparities based on the selected patient population and SDOH index. Most national studies use zip codes, which encompass a wide distribution of communities, limiting the generalizability of findings.

**Objectives:**

To characterize the distributions of composite SDOH indices for pediatric surgical patients within a national sample of academic children’s hospitals and to assess SDOH index precision in classifying patients at similar levels of disadvantage.

**Design, Setting, and Participants:**

This multicenter retrospective cohort study included patients younger than 18 years who underwent surgery from January 1, 2016, to December 31, 2021, at 8 American College of Surgeons National Surgical Quality Improvement Program Pediatric children’s hospitals. Data were analyzed November 15, 2023, to September 25, 2024.

**Exposures:**

Exposures included the Social Vulnerability Index (SVI), Area Deprivation Index (ADI), and Child Opportunity Index (COI), which are composite scores of SDOH factors within a geographic area. A standardized, high-fidelity process was developed to link individual patients to SDOH indices at the US census tract and census block group level.

**Main Outcomes and Measures:**

The primary outcome was composite SDOH index distribution, which was assessed using interrater reliability scores. Substantial agreement was defined as a Cohen κ statistic higher than 0.60.

**Results:**

Of 55 865 included patients, 54.6% were male; 34.8% were infants and toddlers (0-3 years of age), 39.0% were school age (4-12 years), and 26.2% were adolescents (13-17 years). A total of 3468 patients (6.2%) could not be matched to either an SVI, ADI, or COI. Patients with missing geocodes were more likely to be Hispanic (20.1% vs 17.1%; *P* < .001) and have Medicaid insurance (48.1% vs 44.6%; *P* < .001) compared with patients with addresses that could be geocoded. With all institutions grouped, SDOH indices showed only minor variations. However, within each institution and among institutions, indices varied considerably, especially the ADI. Indices had low-to-fair interrater reliability within institutions (κ range, 0.15-0.33), indicating that each index classified individual patients differently according to disadvantage.

**Conclusions and Relevance:**

In this multicenter retrospective cohort study of 55 865 pediatric surgical patients, 3 different composite measures of SDOH classified disadvantage for individual patients differently. The SDOH index components should be understood and carefully considered prior to inclusion of a composite measure in the analysis of children’s surgical outcomes.

## Key Points

**Question:**

Does the distribution of area-based social determinants of health (SDOH) for children undergoing surgical procedures vary among children’s hospitals?

**Findings:**

In this cohort study of 55 865 pediatric patients, SDOH index scores varied considerably, both within each institution and among institutions. Indices also had low to fair interrater reliability, indicating that each index classified the disadvantage of individual patients differently, even within the same institution.

**Meaning:**

Findings of this cohort study suggest that researchers should carefully consider the population and health outcomes of interest when selecting a composite SDOH index to assess the association of SDOH with health care outcomes.

## Teaser

This cohort study characterizes the distribution of area-based social determinates of health (SDOH) indices for pediatric surgical patients in a national sample of academic children’s hospitals and evaluates SDOH index precision in classifying patients at similar levels of disadvantage.

## Introduction

Social determinants of health (SDOH) impact the health care needs and outcomes of pediatric surgical patients in the United States.^1,2,3,4^ SDOH can be divided into 5 primary areas: neighborhood and built environment, health care access and quality, economic stability, education access and quality, and social and community context.^5^ To address the complexity of SDOH and enable analysis of their impact, several composite area-based indices have been developed, including the Area Deprivation Index (ADI), Social Vulnerability Index (SVI), and Child Opportunity Index (COI).^6,7,8^ The associations between patient demographics, clinical outcomes, and SDOH using these indices have been examined in an increasing number of studies.

Although the published literature is growing, the information obtained and conclusions drawn vary significantly according to the examined specific patient population, included index, and the categorizations with the index.^1,9,10^ This variability likely stems from multiple factors, including the granularity of each index and its intended use.^11,12^ SDOH indices map to different sized geographic units, ranging from large zip codes to smaller US census block groups. For example, the population within each zip code may include tens of thousands of individuals and encompass a wide distribution of communities and social circumstances. In contrast, US census tracts contain only 1500 to 8000 people, and census block groups are even smaller (600-3000 people).^13^ The smaller the geographic area, the more consistent and directly relevant the index score is to an individual’s social circumstances. However, because of challenges in sharing fully identifiable data, which is required for US census-based linkage, most national studies use less accurate zip code–level data.^14,15,16^ The use of less granular geographic-based data may attenuate the detection of disparities impacting individual patients and limit the data’s applicability for targeted community-based interventions.

Accounting for more than 40% of pediatric inpatient days despite only comprising less than 5% of all US hospitals, children’s hospitals serve as a critical backbone for pediatric health care in the US.^17^ This low percentage creates geographic gaps in coverage and may exacerbate health disparities due to diminished access to pediatric-specific care. In addition, due to the acuity, resource burden, and emergent nature of many pediatric surgical diseases, challenges with health care access may disproportionately impact pediatric surgical patients. However, the needs of the population served by each children’s hospital and the influence of their contextual circumstances (SDOH) are unknown, limiting the ability of children’s hospitals to respond to individual patients’ needs.

The purpose of this study was to characterize the distributions of composite SDOH indices for pediatric surgical patients in a sample of US academic children’s hospitals and assess the precision of SDOH indices in classifying patients at similar levels of disadvantage. We hypothesized that the distributions of SDOH would vary among institutions. Secondarily, we aimed to develop a standardized approach to geocoding, sharing, and analyzing patient data to increase the fidelity of these processes and to facilitate future comparisons among institutions.

## Methods

### Study Design

We conducted a multicenter retrospective cohort study of pediatric surgical patients at 8 academic children’s hospitals located throughout the continental US. Children included in the study were younger than 18 years of age, underwent surgery between January 1, 2016, and December 31, 2021, and were included in the American College of Surgeons National Surgical Quality Improvement Program (NSQIP) Pediatric database. NSQIP Pediatric is a national registry that includes standardized perioperative and 30-day patient outcome data for a sample of patients who undergo NSQIP Pediatric–eligible procedures. Eligible procedures included those performed by cardiothoracic, neurosurgical, orthopedic, otolaryngologic, pediatric general, urologic, and plastic surgery specialists. The operative log is reviewed on an 8-day cycle.^18,19^ Individual patient addresses were geocoded (described below) and linked to an SVI, ADI, and COI score. The specific indices (SVI, ADI, and COI) were chosen based on a recent systematic literature review that demonstrated frequent use of these indices to examine pediatric surgical outcomes^11^ and linkage to geocoded addresses at either the census tract or block group level. Before data collection, all involved sites obtained ethical approval from their respective institutional review board, and a waiver for the requirement of obtaining informed consent was obtained from each of those boards. The Strengthening the Reporting of Observational Studies in Epidemiology (STROBE) reporting guideline for cohort studies was followed.

### Geocoding Process

Geocoding is the process by which an aspatial locationally descriptive text (ie, postal address) is converted into a valid spatial representation using a predefined process.^20^ To use US census–based indices, patient addresses must undergo geocoding to determine the associated census tract or block group. Unlike zip codes, this requires a fully identifiable dataset, as raw patient addresses are used to assign a corresponding longitude and latitude. In the US, these geocoded data can be converted into corresponding Federal Information Processing Standards codes,^21^ which are assigned by the American National Standards Institute and published by the US census to enable linkage of individual addresses to census hierarchical nested spatial units (described in eAppendix 1 in Supplement 1).^22^

Several common challenges arise for multicenter studies using geocoded data at the census tract or block group level: (1) sharing protected health information between institutions, (2) data preprocessing, and (3) missing or inaccurately coded addresses.

#### Data Sharing

For this study, each institution had differing human participants research policies and requirements regarding the ability to share fully identified data. While a collective multiparty data use agreement was signed between the data coordinating and study sites, 3 of 8 sites could only share a limited dataset. Therefore, before sharing data with the coordinating site, each of these 3 sites geocoded patient addresses, linked them to the SDOH indices, and subsequently deidentified their data. To facilitate this process, the following geocoding process was developed to allow for consistent geocoding across sites and to decrease missingness.

#### Data Preprocessing and Geocoding

Each patient’s home address was extracted from the NSQIP Pediatric institutional dataset. Raw postal address descriptors were converted into a standardized format using a custom-written algorithm (Stata, version 17; StataCorp LLC).

For 5 sites that allowed the sharing of fully identifiable data, addresses were converted into latitude and longitude coordinates using Decentralized Geomarker Assessment for Multi-Site Studies and Docker Desktop, a standalone, container-based application that runs locally on the user’s computer. Addresses unable to be geocoded in this initial step were manually cleaned and converted into latitude and longitude coordinates with the Google Maps Geocoding application programming interface.^23^ Latitude and longitude coordinates were subsequently converted to Federal Information Processing Standards codes, which were then used to assign an ADI, COI, and SVI to each patient using each publicly available database.

For 3 sites that performed geocoding locally, the exact process of geocoding addresses varied slightly. However, efforts were made to limit missingness by sharing best practices for geocoding processes among all sites.

### Variables

#### SDOH Area-Based Indices

**Figure 1.**
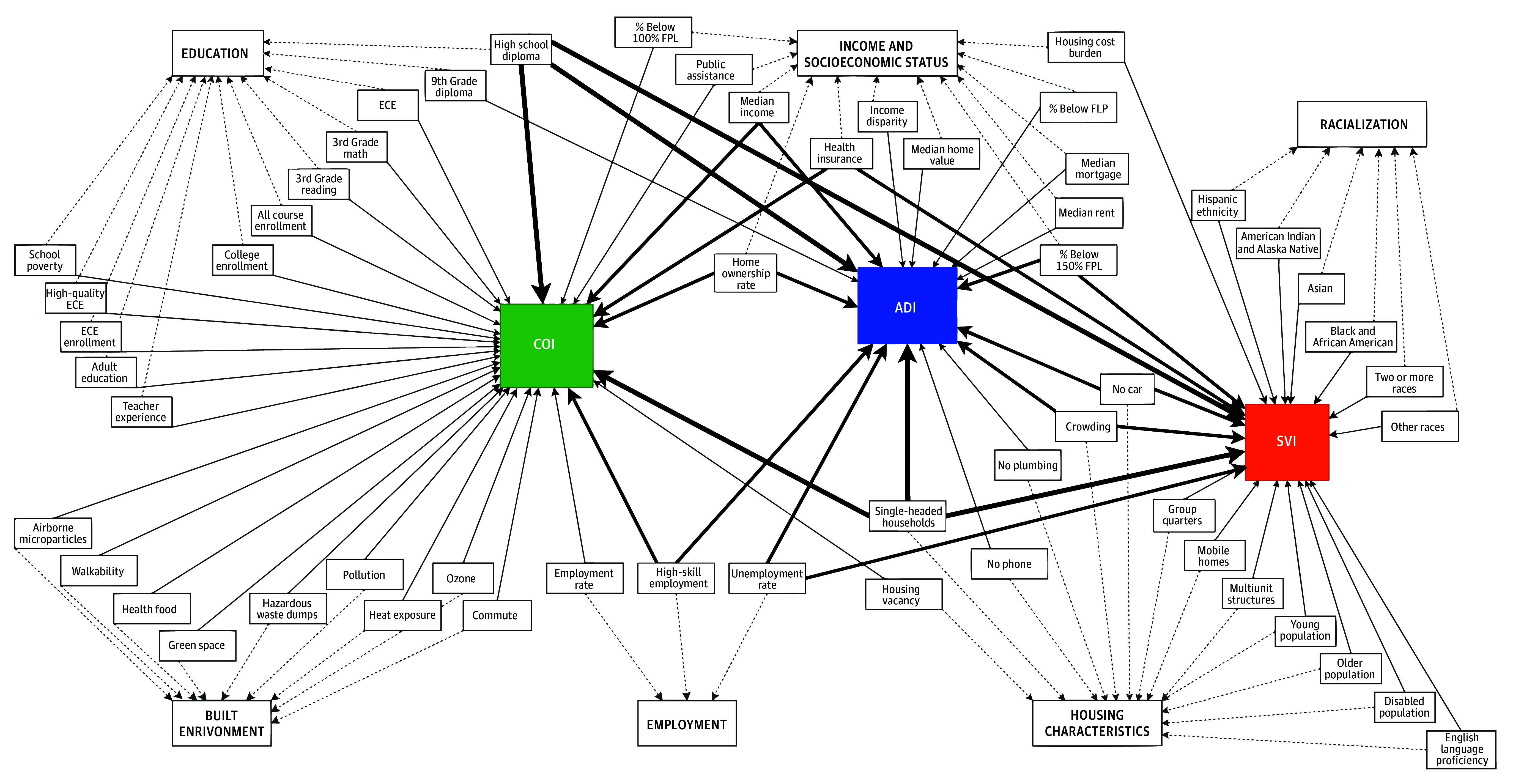
Components of Area-Based Indices for Social Determinants of Health Each index—the Area Deprivation Index (ADI), Social Vulnerability Index (SVI), and Child Opportunity Index (COI)—uses a variable number of components in its calculation. These components can be divided into categories (shown with dotted connecting lines): education, income & socioeconomic status, race and ethnicity as a single variable, built environment, employment, and housing characteristics. Solid lines connect each index to its constituent components. The weight of the solid line indicates how many indices share that constituent component (eg, high school diploma is shared by all 3 indices). Definitions and sources of each component are given in eAppendix 2 in Supplement 1. AP indicates advanced placement; ECE, early childhood education; and FPL, federal poverty level.

Each index uses national data from a variety of sources to create a composite score describing the level of advantage or disadvantage within an area (Figure 1). For the ADI, the national percentile was used, given the national distribution of the study sites. We used COI, version 2.0, for this study.^24^ eAppendix 2 and eTable 1 in Supplement 1 provide detailed descriptions of each index, with a notation of the source data for each component variable included within the composite index. To assess classification consistency (or precision), indices were divided into quintiles according to the SDOH index percentile score (0-20 = very disadvantaged; 21-40 = disadvantaged; 41-60 = average; 61-80 = advantaged; and 81-100 = very advantaged). To align index directionality with the ADI and SVI, the COI was reversed (101 − COI) so that higher scores represented patients from more disadvantaged areas, and lower scores represented patients from more advantaged areas.

#### Other Variables

Demographic variables abstracted from NSQIP Pediatric included age, sex, race and ethnicity, English language preference, presence of a comorbidity, weight, height, insurance type, and the American Society of Anesthesiologists (ASA) Physical Status classification. Age was divided into 3 groups: infant and toddler (0-3 years), school age (4-12 years), and adolescent (13-17 years). Race and ethnicity were assessed because of the previously described associations between race and ethnicity and perioperative disparities and were combined into a single variable. The NSQIP Pediatric collects both race and ethnicity based on an institution’s process (ie, race and ethnicity may be self-assigned or assigned according to internal practices). The race variable included the following options: American Indian or Alaska Native, Asian, Black or African American, Native Hawaiian or Other Pacific Islander, White, other race, and unknown or not reported. The ethnicity variable consisted of Hispanic ethnicity–yes, Hispanic ethnicity–no, and unknown. After combining the race and ethnicity variables into a single variable, the following categories were created: Asian (Non-Hispanic and Asian); Black or African American (Non-Hispanic and Black or African American); Hispanic, White; multiracial or other race (Hispanic and American Indian or Alaska Native; Hispanic and Asian; Hispanic and Black or African American; Hispanic and Native Hawaiian or Other Pacific Islander; Hispanic and some other race; Non-Hispanic and American Indian or Alaska Native; Non-Hispanic and Native Hawaiian or Other Pacific Islander; Non-Hispanic and some other race); Non-Hispanic, White; and missing or not reported. Any individual who selected unknown race and Hispanic ethnicity–yes was included in the group White, Hispanic.

Comorbidity was defined through the combination of standardized preoperative comorbidities listed in NSQIP Pediatric. Obesity was defined as any person 2 to17 years of age with a body mass index at the 95th percentile or higher for age and sex.^25^ Any child younger than 2 years was defined as having obesity if their weight (kg) for age and sex was at the 95th percentile or higher.^25^ Insurance type was divided into 5 categories: commercial, Medicaid, Medicare, self-pay, and other or unknown. ASA physical status classification I (normal healthy patient) and ASA II (mild systemic disease) were kept as individual categories. We collapsed ASA III or higher into a single category, including ASA III (severe systemic disease), ASA IV (severe systemic disease, threatening to life), and ASA V (moribund, not expected to survive without the operation).

### Statistical Analysis

Stata, version 17 (StataCorp LLC), was used to conduct all analyses. The characteristics of participants who could or could not be geocoded were examined using descriptive statistics. Categorical variables were compared using χ^2^ tests. Continuous variables were assessed with *t* tests and Wilcoxon rank sum tests, as appropriate. All index distributions were compared across sites. Index consistency and correlation in classifying patients at similar levels of disadvantage (ie, by quintile) were assessed using interrater reliability (IRR) and Spearman correlation coefficient analyses. For the IRR analysis, each index was considered a rater, and these comparisons occurred at the individual hospital level, such that the IRR represented the precision of each index in classifying individual patients into the same quintiles of advantage. Substantial agreement was defined as a Cohen κ statistic higher than 0.60. Correlation was also assessed at the individual hospital level. Hypothesis tests were 2-sided, and statistical significance was set at *P* < .05. Data were analyzed November 15, 2023, to September 25, 2024.

## Results

A total of 59 787 children were identified from 8 children’s hospitals during the study period. After excluding 3922 patients who lacked address documentation, the final cohort included 55 865 patients. Of these patients, 54.6% were male and 45.4% were female; 34.8% were infants and toddlers, 39.0% were school age, and 26.2% were adolescents; 38.7% were from historically racial and ethnic minoritized groups (5.1% Asian; 13.7% Black or African American; 17.3% Hispanic, White; 2.6% Multiracial or other race); 54.3% were Non-Hispanic, White; 7.0% had no reported race or ethnicity; 44.0% had comorbidities; and 14.5% had obesity (Table 1).

**Table 1.** Demographic Characteristics for All Patients, Patients With No Missing Data (Geocoded), and Patients With Missing SDOH Data Due to Inability to Geocode or to Link to SDOH Indices (Not Geocoded)

Characteristic	Patients, No. (%)			*P* value^a^
	Total (n = 55 865)	Geocoded (n = 52 397)	Not geocoded (n = 3468)	
Age at surgery				
Infant and toddler (0-3 y)	19 455 (34.8)	18 180 (34.7)	1275 (36.8)	.002
School age (4-12 y)	21 794 (39.0)	20 424 (39.0)	1370 (39.5)	
Adolescent (13-17y)	14 616 (26.2)	13 793 (26.3)	823 (23.7)	
Sex				
Female	25 378 (45.4)	23 803 (45.4)	1575 (45.4)	.83
Male	30 478 (54.6)	28 586 (54.6)	1892 (54.6)	
Missing	9 (0.0)	8 (0.0)	1 (0.0)	
Race and ethnicity				
Asian	2844 (5.1)	2697 (5.1)	147 (4.2)	<.001
Black or African American	7647 (13.7)	7283 (13.9)	364 (10.5)	
Hispanic, White	9657 (17.3)	8960 (17.1)	697 (20.1)	
Multiracial or other race^b^	1466 (2.6)	1319 (2.5)	147 (4.2)	
Non-Hispanic, White	30 359 (54.3)	28 583 (54.6)	1776 (51.2)	
Missing or not reported	3892 (7.0)	3555 (6.8)	337 (9.7)	
English language preferred				
No	3044 (5.4)	2843 (5.4)	201 (5.8)	<.001
Yes	26 391 (47.2)	24 985 (47.7)	1406 (40.5)	
Missing	26 430 (47.3)	24 569 (46.9)	1861 (53.7)	
Comorbidity				
No	31 260 (56.0)	29 452 (56.2)	1808 (52.1)	<.001
Yes	24 598 (44.0)	22 940 (43.8)	1658 (47.8)	
Missing	7 (0.0)	5 (0.0)	2 (0.1)	
Obesity				
No	41 620 (74.5)	39 047 (74.5)	2573 (74.2)	.13
Yes	8101 (14.5)	7564 (14.4)	537 (15.5)	
Missing	6144 (11.0)	5786 (11.0)	358 (10.3)	
Insurance type				
Commercial	21 999 (39.4)	20 822 (39.7)	1177 (33.9)	<.001
Medicaid	25 039 (44.8)	23 372 (44.6)	1667 (48.1)	
Medicare	607 (1.1)	550 (1.0)	57 (1.6)	
Self-pay	1848 (3.3)	1788 (3.4)	60 (1.7)	
Other or unknown	6372 (11.4)	5865 (11.2)	507 (14.6)	
ASA Physical Status classification				
ASA I (normal healthy patient)	14 048 (25.1)	13 203 (25.2)	845 (24.4)	.48
ASA II (mild systemic disease)	24 466 (43.8)	22 959 (43.8)	1507 (43.5)	
ASA ≥III (severe systemic disease to moribund)	17 288 (30.9)	16 176 (30.9)	1112 (32.1)	
Missing	63 (0.1)	59 (0.1)	4 (0.1)	

Abbreviations: ASA, American Society of Anesthesiologists; SDOH, social determinants of health.

^a^ Comparisons were between the geocoded and not geocoded groups.

^b^ Multiracial or other race includes the following categories: Hispanic and American Indian or Alaska Native; Hispanic and Asian; Hispanic and Black or African American; Hispanic and Native Hawaiian or Other Pacific Islander; Hispanic and some other race; Non-Hispanic and American Indian or Alaska Native; Non-Hispanic and Native Hawaiian or Other Pacific Islander; Non-Hispanic and some other race.

Of the 5 hospitals that contributed identifiable data, 1809 addresses (5.5%) were unable to be geocoded following first-pass geocoding, with many of the missing addresses noted to be imprecise. The second pass successfully geocoded 533 imprecise addresses (76.0%), reducing missingness to 3.9% (n = 1276). The remaining missingness was largely due to post office (PO) boxes (938 [2.9%]), empty addresses (179 [0.05%]), or international or imprecise addresses (161 [0.05%]). Once linked to SDOH indices, 287 addresses (0.9%) were suppressed by ADI, 0 (were not found in the SVI database, and 760 (2.3%) were not found in the COI database. These data were combined with the 3 sites that internally geocoded their data. eTable 2 in Supplement 1 provides hospital demographics and missingness at each site.

**Figure 2.**
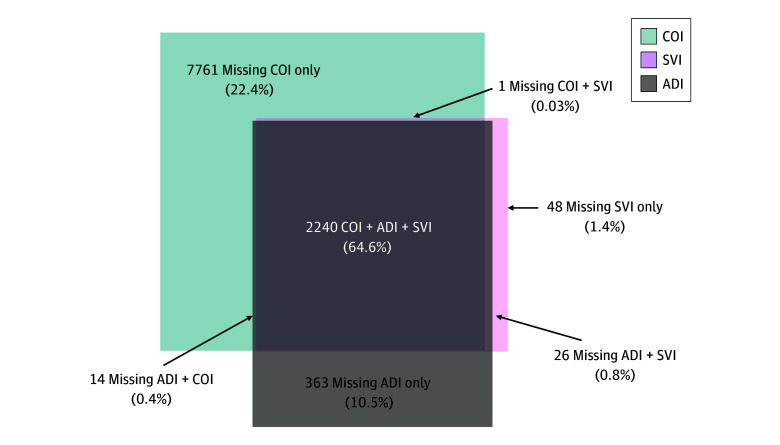
Distribution of Missingness for Social Determinants of Health Indices ADI indicates Area Deprivation Index; COI, Child Opportunity Index; and SVI, Social Vulnerability Index.

In total (all 8 sites), 3468 patients (6.2%) could not be linked to an SVI, ADI, or COI. Of these patients, 2240 (4.1%) could not be linked to any index, a total of 2315 (4.2%) were missing the SVI, 2643 (4.8%) were missing the ADI, and 3031 (5.5%) were missing the COI (Figure 2). Patients with missing data were compared with patients with no missing data (Table 1). The distributions of age at surgery, race and ethnicity as a single variable, and insurance type differed significantly by missing data status (Table 1), with those missing data having a higher proportion of infants and toddlers (nonmissing, 34.7% vs missing, 36.8%), Hispanic, White (17.1% vs 20.1%) and Medicaid insurance (44.6% vs 48.1%).

**Figure 3.**
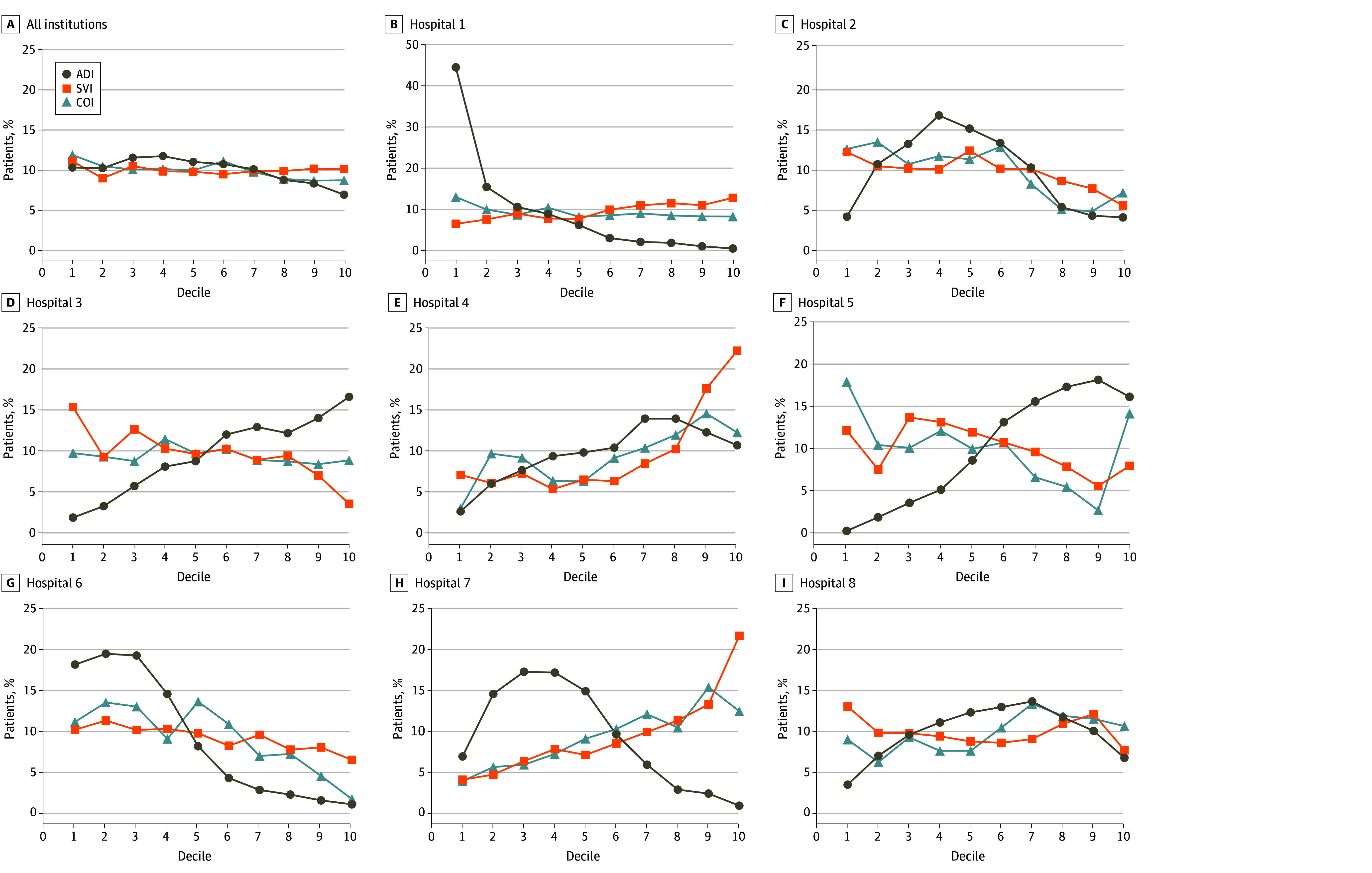
Distribution of Indices for Social Determinants of Health Among 8 Children’s Hospitals For each index, patients were allocated to deciles, and the subsequent distributions were plotted. To align index directionality, the Child Opportunity Index (COI) was reversed (101 − COI). ADI indicates Area Deprivation Index; and SVI, Social Vulnerability Index.

When all institutions were grouped, the indices showed only minor variations. However, within each institution and among institutions, each index varied considerably, especially the ADI (Figure 3). To assess the precision of each index in classifying patients at similar levels of disadvantage, the IRR between indices was calculated for each institution. Indices had low to fair IRR values (κ range, 0.15-0.33), indicating that, within the same institution, each SDOH index classified patients as having different levels of advantage (Table 2). When comparing individual indices, the ADI was the least consistent with the other 2 indices, with agreement ranging from none to fair (κ range, 0.03-0.34) (eTable 3 in Supplement 1). The COI and SVI were more consistent with each other, with fair agreement (κ range, 0.25-0.37) (eTable 3 in Supplement 1). Correlation was moderate between the ADI and SVI (Spearman ρ range, 0.51-0.72), moderate to strong for the ADI and COI (Spearman ρ range, 0.62-0.77), and strong for the COI and SVI (Spearman ρ range, 0.71-0.80) (eTable 4 in Supplement 1).

**Table 2.** Interrater Reliability of All 3 Composite SDOH Indices for Classifying Patients With Respect to Quintile^a^

Hospital site	Agreement, %	Cohen κ Statistic (95% CI)
1	31.5	0.15 (0.14-0.16)
2	44.6	0.30 (0.30-0.32)
3	37.0	0.22 (0.21-0.23)
4	47.3	0.33 (0.32-0.34)
5	33.5	0.18 (0.17-0.19)
6	39.5	0.22 (0.21-0.23)
7	30.9	0.16 (0.15-0.16)
8	44.1	0.30 (0.29-0.31)

Abbreviation: SDOH, social determinants of health.

^a^ For each index, patients were allocated to quintiles, and the interrater reliability between indices (for classifying each patient into the same or different quintile) was calculated.

## Discussion

This cohort study characterized the distributions of composite SDOH indices for pediatric surgical patients in a geographically diverse national sample of academic children’s hospitals. We observed that the distribution of SDOH indices varied not only among institutions but also within an institution, showing variable precision within SDOH indices for classifying patients at similar levels of disadvantage. These results can guide future investigations using area-based SDOH indices to evaluate and address disparities in pediatric surgical outcomes.

Our finding that SDOH index scores varied widely within and among institutions highlights the importance of thoughtful index selection. Some of the differences in this multi-institutional study are likely derived from the original use cases and the technical details underpinning the design of each score. For example, the COI seeks to capture resources that specifically impact child health and development, with outcomes and weights assigned to the constituent components of the score according to their association with health outcomes.^7,12,26^ Conversely, the SVI was designed to assess community vulnerability to disasters and relies on expert consensus for selecting component variables.^6,12^ Although the ADI is the most commonly used index in the pediatric surgical population^11^ and was designed to inform health delivery and policy, it has been criticized for not standardizing component measures prior to calculating index scores.^27^ Consequently, the ADI heavily weights income and home values over its other constituent components.^27,28^ This weighting may explain why ADI shows an almost inverse relationship to the COI and SVI in some institutions, such as hospital 5 and hospital 7 (located in states with higher housing costs; Figure 3). These variations lead to classification of children at these sites as more advantaged, compared with the national population, whereas if adjusted for costs of housing and living, the classified level of advantage would be considerably lower. Some indices had more missingness in certain regions than others (eTable 2 in Supplement 1), and these differences in geographic coverage may impact the quality of SDOH data obtained for patients within a region. Variations in missing data among institutions may also contribute to the inter-institutional variability in SDOH indices. Researchers should carefully consider the potential biases that may be introduced by a given index based on methodology and regional penetrance when choosing an SDOH index. Using more than 1 index, particularly if assessing for global SDOH to identify comprehensive associations across populations, should also be considered.

The inconsistency of SDOH indices in classifying patients at the same level of advantage cannot solely be attributed to the components of the index. While the lowest IRRs and correlations were noted with the ADI, the κ statistics for the COI and SVI remained lower than 0.40 at every site, suggesting at most fair agreement of these indices, with strong but not very strong correlation. Allocating patients to SDOH index quintiles may have created false dichotomies that contributed to this imprecision by separating closely related patients who fell on either side of a dichotomy line and thus exacerbated differences between categories. Therefore, when sample size allows, maintaining index scores in a continuous format to avoid problems associated with binning of data should also be considered.

Our results also highlight the risk of introducing bias through geocoding processes. Hispanic patients and patients with Medicaid insurance were significantly more likely to be excluded through geocoding. Previous studies have shown that populations with high geocoding missingness are more likely to be rural^29^ and have a PO box as the primary address in the health record. Using a PO box as a primary address may be due to residence in an area without a standard street address, mobile home, houselessness, migrant work, or undocumented status, which raises concern that excluded populations may include individuals with the least advantage.^30^ Few efficient methods exist to reclassify these patients, and simple solutions, such as using the PO box zip code, may lead to inaccurate geographic designation for over 80% of PO box holders.^31^ Thus, the best solution may be to consistently assess the demographics of persons who are or are not geocoded to guide sensitivity analyses and consideration of bias in any study examining SDOH indices.

Finally, we found that standardized high-fidelity geocoding was feasible and straightforward, resulting in minimal dataset missingness. Multicenter studies commonly use zip codes, as they can be shared more easily and require no geocoding. However, zip code–based studies are limited due to the imprecision of the geographic unit used, which limits the ability to detect and thereby mitigate disparities, as the areas of analysis are large, heterogeneous, and change frequently. In contrast, census tracts and census block groups are much smaller geographic designations that are more relevant to an individual’s social circumstances.^13^ Given the recent growth in health disparities research, there is an increasing need for health systems to adopt geocoding processes and to develop standardized policies for using and sharing geocoded data. Broader adoption of standardized geocoding processes, such as those described in this article, will facilitate multi-institutional collaboration and data sharing while protecting personal health information. This adoption will enable researchers to link health datasets and work toward the identification of modifiable factors associated with health disparities.

### Limitations

While this large multicenter study represents a critical step forward to enable examination of how SDOH are associated with health outcomes in a pediatric surgical population, there are limitations to consider. The retrospective nature of this database study limited our ability to collect SDOH data directly from individual patients and thereby validate the composite SDOH scores within our patient populations. Inconsistent documentation was noted in the capture of patient-preferred language, with some sites having complete data with low missingness, while other sites had almost no language documentation; the 47% overall missingness of this variable limited the assessment of geocoding fidelity among non-English–speaking patients. This study also overlapped temporally with the 2020 US Census. At the time of this study, both the ADI and SVI had been updated to the 2020 census for linkage, but COI, version 3.0, had yet to be released, and COI, version 2.0, was linked based on 2010 geographic census designations. These changes and the potential for shifting demographics over time may have contributed to the poor IRR observed in this study. In addition, a lack of representation from intermountain and southwest hospitals may limit the generalizability of the data. All participating hospitals were tertiary referral centers, and it is difficult to know if geocoding fidelity would be maintained at the community level. Consistent data collection of SDOH variables from patients and integrating geocoding processes at the database level may help address these challenges.

## Conclusions

In this cohort study of 55 865 patients, SDOH indices varied both among and within institutions. These variations highlight the critical importance of carefully considering the population and health outcomes of interest when selecting an SDOH index for use and maintaining indices as continuous variables when possible. The integration of area-based composite SDOH indices into analyses of health outcomes presents a vital opportunity to understand and tackle the root causes of health inequity. Healthcare organizations and quality programs, such as the NSQIP Pediatric program, should consider integrating standardized geocoding processes to facilitate the incorporation of SDOH indices into analyses. When evaluating the impact of SDOH broadly across populations, we recommend using 2 or more SDOH indices to identify consistent associations.

## Notes

Supplement 1. **eAppendix 1.** Understanding geographic designations & geocoding best practices **eAppendix 2.** Understanding area-based indices **eTable 1.** Component variables for each index **eTable 2.** Participating hospital demographics **eTable 3.** Interrater reliability of SDOH indices classifying patients within the same quintile with 2-index comparisons **eTable 4.** Spearman’s correlation coefficients of SDOH indices classifying patients within the same quintile with 2-index comparisons

Supplement 2.
Data Sharing Statement
