# Supplementary material for: Comparative Analysis of Indices for Social Determinants of Health in Pediatric Surgical Populations
Source: JAMA Netw Open. 2024 Dec 10;7(12):e2449672. doi: 10.1001/jamanetworkopen.2024.49672 (PMC11632545; doi:10.1001/jamanetworkopen.2024.49672)
Supplement: Supplement 1. — eAppendix 1. Understanding geographic designations & geocoding best practices eAppendix 2. Understanding area-based indices eTable 1. Component variables for each index eTable 2. Participating hospital demographics eTable 3. Interrater reliability of SDOH indices classifying patients within the same quintile with 2-index comparisons eTable 4. Spearman’s correlation coefficients of SDOH indices classifying patients within the same quintile with 2-index comparisons [file jamanetwopen-e2449672-s001.pdf]

Supplementary Online Content

Stephens CQ, Yap A, Vu L, et al. Comparative analysis of indices for social determinants of health in pediatric surgical populations. *JAMA Netw Open*. 2024;7(12):e2449672. doi:10.1001/jamanetworkopen.2024.49672

eAppendix 1. Understanding geographic designations & geocoding best practices
eAppendix 2. Understanding area-based indices
eTable 1. Component variables for each index
eTable 2. Participating hospital demographics
eTable 3. Inter-rater reliability of SDoH indices classifying patients within the same quintile with 2-index comparisons
eTable 4. Spearman's correlation coefficients of SDoH indices classifying patients within the same quintile with 2-index comparisons
eReferences.

This supplementary material has been provided by the authors to give readers additional information about their work.

© 2024 Stephens CQ et al. *JAMA Network Open*.

eAppendix 1. Understanding Geographic designations & Geocoding Best Practices

ZIP Codes: ZIP codes and ZIP code tabulation areas (ZCTAs) are frequently represented as polygonal areas for analysis, but they lack standardization and are highly dynamic in structure.[1] Therefore, both units can be problematic due to two major issues: 1. Spatiotemporal mismatch, and 2. Lack of granularity. Spatiotemporal mismatch occurs when data collected in both space and time do not coincide.[1] For example, in the current study, address data were collected for patients who presented to care between 1/1/2016 and 12/21/2021. However, ADI is derived from the US Census American Community Survey (ACS) Five Year Estimates, with the most recent ADI using 2020 ACS data. As ZIP codes can be revised at any time, there is a strong potential for spatiotemporal mismatch in these data. In addition, since ZIP codes only exist to expedite the delivery of mail,[2] the US Postal Service (USPS) does not attempt to optimize the size or population allocation of ZIP codes. Thus, ZIP codes range in size from a single building to thousands of square miles and contain populations that vary widely within those spatial boundaries.

US Census Bureau: The U.S. Census Bureau developed hierarchically nested spatial units (census blocks, block groups, tracts, counties, etc.)[3] with alterations tracked in decennial surveys, allowing for more accurate longitudinal analyses.[4] Rather than being based on delivery routes and/or landmarks, these units rely on spatial divisions that are more relevant in a societal context, such as topography, land survey systems, details and density of urban or rural development, and the presence of important community-specific boundaries, such as American Indian reservations.[5] These units also change less frequency from census to census. In order to generate these units, individual addresses must be matched to the Federal Information Processing Standards (FIPS) code assigned by the Census Bureau to the respective address.

Geocoding Best Practices: In 2008, the North American Association of Central Cancer Registries published "A Geocoding Best Practices Guide," describing several methods of geocoding and establishing best practices for health research based on patient registries.[6] Similarly, a 2014 short communication in the journal *Epidemiology* described a method using the Google Maps application programming interface (API) and the R software environment to perform geocoding.[7] Furthermore, the Public Health Disparities Geocoding Project, run by the Harvard T. H. Chan School of Public Health, currently offers a training manual for geocoding and using area-based socioeconomic measures to overcome the absence of socioeconomic data in most US health records.[8]

© 2024 Stephens CQ et al. *JAMA Network Open*.

eAppendix 2. Understanding Area-Based Indices

Area Deprivation Index

The Area Deprivation Index (ADI) was designed in 2003 by the Health Resources & Services Administration (HRSA) with the goal of informing healthcare delivery and policy, particularly within disadvantaged areas.[9] Originally linked to mortality at the census tract level,[9] ADI has since been refined and validated to the Census Block Group level. ADI includes 17 measures of social risk within the domains of income, education, employment, and housing quality.[10] ADI data is accessible for use through the Neighborhood Atlas.[10] ADI ranks census block groups (neighborhoods) as national percentiles from 1 (least disadvantaged) to 100 (most disadvantaged). The data are derived from the American Community Survey (ACS) Five Year Estimates. Therefore, the rankings based upon this survey data are subject to the same limitations as five-year averages, including limited accounting for undocumented-immigrant populations.[10] ADI is also available in deciles from 1 (least disadvantaged) to 10 (most disadvantaged) for each individual state. For ADI, certain geocodes are suppressed for the following reasons: (1) low population and/or housing (<100 people or <30 housing units); (2) high group quarters (>33% of the population living in group quarters); or (3) questionable data integrity (missing data in source ACS data).

Child Opportunity Index

The Child Opportunity Index (COI) was developed to capture resources and conditions that matter specifically for children's health, development, and long-term outcomes, such as health and socioeconomic mobility.[11,12] Uniquely, COI was designed to capture neighborhood resources that facilitate healthy child development, not as an index of concentrated disadvantage or vulnerability.[13] While COI 3.0 has recently been released, COI 2.0 was used for this study. It includes 39 measures over three domains (education, health and environment, and social and economic) and is reported on a scale from 1 (lowest opportunity) to 100 (highest opportunity), representing true national percentiles at the US Census tract geographic level. Each measure of social risk is individually weighted based upon data from the Opportunity Atlas, RWJF 500 Cities Project and the CDC.[14] These percentiles can then be further categorized into five levels, ranging from "very low" to "very high" opportunity.

Social Vulnerability Index

The Social Vulnerability Index (SVI) was developed by the United States Centers for Disease Control and Prevention (CDC)/Agency for Toxic Substances and Disease Registry (ATSDR) to identify communities that may need assistance in the setting of a hazardous event, such as natural or human-caused disasters, including disease outbreaks.[15] For example, SVI has been used to estimate the amount of supplies (i.e., food, water, medicine) needed during a disaster, to help allocate emergency preparedness funding based on community need, to decide how many emergency personnel are required to assist people during a disaster, and to identify areas in need of emergency shelters.[16] SVI uses variables from the US Census and the ACS to identify at-risk groups by ranking all US census tracts or counties from 0.0 (least vulnerable) to 1.0 (most vulnerable).[16] SVI rankings are available at both the state and national levels, and use 14 social factors, which can be grouped into four themes: socioeconomic status, household composition and disability, minority status and language, and housing type and transportation.[16] Advantages of the SVI include that SVI is updated every two years, which is more frequent than the other two included metrics and the ability to drill down to each of the four composite themes. However, while this index has been associated with poor surgical outcomes in the pediatric populations,[17] not all included measures are validated as relevant to pediatric health or healthcare.

© 2024 Stephens CQ et al. *JAMA Network Open*.

eTable 1. Component variables for each index. Colors indicate to which index each component variable belongs. Variable name corresponds to the names shown in Figure 1. Variable definitions are provided, with the source of each variable in parentheses. ADI: Area Deprivation Index; SVI: Social Vulnerability Index; COI: Child Opportunity Index

ADI	SVI	COI	Variable Name	Variable definition (source)
			**EDUCATION**	
■			9TH GRADE EDUCATION	Percent of the block group's population aged ≥ 25 years with < 9 years of education (ACS)
■			HIGH SCHOOL DIPLOMA[a,b]	Percent age ≥ 25 years with greater than or equal to a high school diploma, reversed (ACS)
	■		NO HIGH SCHOOL DIPLOMA[a]	Percent age ≥ 25 years with less than a 12th grade education (including individuals with 12 grades but no diploma). (ACS)
		■	ECE	Early childhood education (ECE); Number of ECE centers within a 5-mile radius (COI data collection from state and federal sources)
		■	HIGH-QUALITY ECE	High-quality ECE centers; Number of NAEYC accredited centers within a 5-mile radius (COI data collection from state and federal sources)
		■	ECE ENROLLMENT	Percent 3- and 4-year-olds enrolled in nursery school, preschool or kindergarten (ACS)
		■	3RD GRADE READING	Third grade reading proficiency; Percent third graders scoring proficient on standardized reading tests, converted to NAEP scale score points (EDFacts, GS and SEDA)
		■	3RD GRADE MATH	Third grade math proficiency; Percent third graders scoring proficient on standardized math tests, converted to NAEP scale score points (EDFacts, GS and SEDA)
		■	HIGH SCHOOL GRADUATION[b]	High school graduation rate; Percent ninth graders graduating from high school on time (EDFacts and GS)
		■	AP COURSE ENROLLMENT	Advanced Placement (AP) course enrollment; Ratio of students enrolled in at least one AP course to the number of 11th and 12th graders (CRDC)
		■	COLLEGE ENROLLMENT	College enrollment in nearby institutions; Percent 18-24-year-olds enrolled in college within 25-mile radius (ACS)
		■	SCHOOL POVERTY	Percent students in elementary schools eligible for free or reduced-price lunches, reversed (NCES, CCD)
		■	TEACHER EXPERIENCE	Percent teachers in their first and second year, reversed (CRDC)
		■	ADULT EDUCATION	Adult educational attainment; Percent adults ages 25 and over with a college degree or higher. (ACS)
			**EMPLOYMENT**	
		■	EMPLOYMENT RATE	Percent adults ages 25-54 years who are employed. (ACS)
■	■		UNEMPLOYMENT RATE	Percent of civilian labor force population ≥ 16 years of age unemployed (unemployment rate). Unemployed persons actively seeking work. (ACS)
■		■	HIGH-SKILL EMPLOYMENT	Percent employed persons ≥16 years of age employed in management, business, financial, computer, engineering, science, education, legal, community service, health care practitioner, health technology, arts and media occupations. (ACS)
			**BUILT ENVIRONMENT**	
		■	AIRBORNE MICROPARTICLES	Mean estimated microparticle (PM2.5) concentration, reversed (CDC)
		■	WALKABILITY	EPA Walkability Index (EPA)
		■	HEALTH FOOD	Access to healthy food; Percent households without a car located further than a half-mile from the nearest supermarket, reversed (USDA)
		■	GREEN SPACE	Access to green space; Percent impenetrable surface areas such as rooftops, roads or parking lots, reversed (CDC)
		■	HAZARDOUS WASTE DUMPS	Hazardous waste dump sites; Average number of Superfund sites within a 2-mile radius, reversed (EPA)
		■	POLLUTION	Industrial pollutants in air, water, or soil; Index of toxic chemicals released by industrial facilities, reversed (EPA)
		■	HEAT EXPOSURE	Extreme heat exposure; Summer days with maximum temperature above 90F, reversed (CDC)
		■	OZONE	Ozone concentration; Mean estimated 8-hour average ozone concentration, reversed (EPA)
		■	COMMUTE	Commute duration; Percent workers commuting more than one hour one way, reversed (ACS)

© 2024 Stephens CQ et al. *JAMA Network Open*.

ADI	SVI	COI	Variable Name	Variable definition (source)
**HOUSING CHARACTERISTICS**				
		■	HOUSING VACANCY	Housing vacancy rate; Percent housing units that are vacant, reversed (ACS)
	■		YOUNG POPULATION	Aged 17 & Younger
	■		DISABLED POPULATION	Civilian with a Disability
	■		ENGLISH LANGUAGE PROFICIENCY	English Language Proficiency
	■	■	OLDER POPULATION	Aged 65 & Older
■	■	■	SINGLE-HEADED HOUSEHOLDS	Percent of single-parent households with children < 18 years of age (ACS)
■	■		NO CAR	Percent of occupied housing units without a motor vehicle (ACS)
■			NO PHONE	Percent of occupied housing units without a telephone (ACS)
■			NO PLUMBING	Percent of occupied housing units without complete plumbing (ACS)
■	■		CROWDING	Percent of occupied housing units with more than one person per room (ACS)
	■		MULTI-UNIT STRUCTURES	Multi-Unit Structures
	■		MOBILE HOMES	Mobile Homes
	■		GROUP QUARTERS	Group quarters
**INCOME & SOCIOECONOMIC STATUS**				
	■		NO HEALTH INSURANCE[a]	Percent individuals ages 0-64 years with NO health insurance coverage, reversed (ACS)
		■	HEALTH INSURANCE[a]	Percent individuals ages 0-64 years with health insurance coverage (ACS)
■		■	MEDIAN INCOME	Median family/household income (ACS)
■			INCOME DISPARITY	Income disparity (ACS)
■			% BELOW FPL	Poverty rate; Percent of families below the poverty level (ACS)
		■	% BELOW 100FPL	Poverty rate; Percent individuals living in households with incomes below 100% of the federal poverty threshold, reversed (ACS)
■	■		% BELOW 150FPL	Poverty rate; Percent of population below 150% of the poverty threshold (ACS)
■			MEDIAN HOME VALUE	Median home value (ACS)
■			MEDIAN RENT	Median gross rent (ACS)
■			MEDIAN MORTGAGE	Median monthly mortgage (ACS)
■		■	HOME OWNERSHIP RATE	Percent owner-occupied housing units (home ownership rate) (ACS)
	■		HOUSING COST BURDEN	Households that spend 30% or more of annual income on housing costs
		■	PUBLIC ASSISTANCE	Public assistance rate; Percent households receiving cash public assistance or Food Stamps/Supplemental Nutrition Assistance Program, reversed (ACS)
**RACIALIZATION**				
	■		HISPANIC ETHNICITY	Hispanic or Latino (of any race);
	■		BLACK & AFRICAN AMERICAN	Black and African American, Not Hispanic or Latino;
	■		AMERICAN INDIAN & ALASKA NATIVE	American Indian and Alaska Native, Not Hispanic or Latino;
	■		ASIAN	Asian, Not Hispanic or Latino; Native Hawaiian and Other Pacific Islander, Not Hispanic or Latino;
	■		TWO OR MORE RACES	Two or More Races, Not Hispanic or Latino;
	■		OTHER RACES	Other Races, Not Hispanic or Latino

Abbreviations: ACS: Data from the American Community Survey; ADI: Area Deprivation Index; AP: Advanced Placement; CCD: NCES Common Core of Data; CDC: Centers for Disease Control and Prevention; COI: Child Opportunity Index; CRDC: Civil Rights Data Collection; ECE: Early childhood Education; EDFacts: a U.S. Department of Education (ED) initiative to collect, analyze, and promote the use of high-quality, pre-kindergarten through grade 12 data; EPA: Environmental Protection Agency; GS: GreatSchools; NAEP: National Assessment of Educational Progress; NAEYC: The National Association for the Education of Young Children; NCES: National Center for Education Statistics; SEDA: Stanford Education Data Archive; SVI: Social Vulnerability Index; USDA: United States Department of Agriculture

[a] Similar, but inverse, variable. Noted on figure as a single variable (HIGH SCHOOL DIPLOMA or HEALTH INSURANCE).

[b] Similar variables, but calculated differently and obtained from different sources. Noted on figure as a single variable (HIGH SCHOOL DIPLOMA).

© 2024 Stephens CQ et al. *JAMA Network Open*.

eTable 2. Participating hospital demographics

	No.	US Region	Bed Size, No.	Annual pediatric admissions, No.	Annual pediatric surgeries performed, No.	% study population within 100 miles, No. (%)	Any Missing Index, No. (%)	Missing SVI, No. (%)	Missing ADI, No. (%)	Missing COI, No. (%)
Hospital 1	5,045	West	180	5,870	7,200	3,675 (78)	355 (7.0)	243 (4.8)	269 (5.3)	331 (6.5)
Hospital 2[a]	8,378	Northeast / Mid-Atlantic	205	7,590	14,040	7,302 (93)	342 (4.1)	296 (3.5)	294 (3.5)	248 (3.0)
Hospital 3	8,962	Midwest	455	11,850	10,030	6,324 (75)	657 (7.3)	384 (4.3)	464 (5.2)	584 (6.5)
Hospital 4	5,415	South	330	15,000	7,500	4,705 (94)	448 (8.3)	202 (3.7)	219 (4.0)	431 (8.0)
Hospital 5[a]	6,380	Northeast / Mid-Atlantic	150	7,700	4,155	3,306 (52)	46 (0.7)	19 (0.3)	45 (0.7)	20 (0.3)
Hospital 6[a]	8,225	West	410	14,860	15,150	5,975 (80)	770 (9.4)	724 (8.8)	741 (9.0)	727 (8.8)
Hospital 7	5,499	West	120	7,410	6,160	4,430 (88)	567 (10.3)	322 (5.9)	445 (8.1)	448 (8.1)
Hospital 8	7,961	Southeast	335	19,660	13,665	7,177 (93)	283 (3.6)	125 (1.6)	166 (2.1)	242 (3.0)

Abbreviations: SVI: Social Vulnerability Index; ADI: Area Deprivation Index; COI: Child Opportunity Index

[a] Denotes sites that geocoded their own data internally.

© 2024 Stephens CQ et al. *JAMA Network Open*.

eTable 3. Inter-rater reliability of SDoH indices classifying patients within the same quintile with 2-index comparisons

Area Deprivation Index and Social Vulnerability Index		
**Hospital Site**	**Percent Agreement (%)**	**Cohen's Kappa Statistic (95% Confidence Interval)**
Hospital 1	19.0	0.03 (0.02 - 0.04)
Hospital 2	38.6	0.22 (0.21 - 0.24)
Hospital 3	27.7	0.12 (0.11 - 0.13)
Hospital 4	44.1	0.29 (0.27 - 0.31)
Hospital 5	27.6	0.11 (0.27 - 0.31)
Hospital 6	33.2	0.14 (0.13 - 0.15)
Hospital 7	18.6	0.04 (0.03 - 0.05)
Hospital 8	38.1	0.23 (0.22 - 0.24)
**Area Deprivation Index and Child Opportunity Index**		
**Hospital Site**	**Percent Agreement**	**Cohen's Kappa Statistic (95% Confidence Interval)**
Hospital 1	31.5	0.10 (0.09 - 0.12)
Hospital 2	48.1	0.34 (0.32 - 0.35)
Hospital 3	35.5	0.20 (0.19 - 0.21)
Hospital 4	46.9	0.33 (0.31 - 0.34)
Hospital 5	22.8	0.07 (0.06 - 0.09)
Hospital 6	44.6	0.26 (0.25 - 0.27)
Hospital 7	22.6	0.07 (0.06 - 0.08)
Hospital 8	47.4	0.34 (0.33 - 0.35)
**Child Opportunity Index and Social Vulnerability Index**		
**Hospital Site**	**Percent Agreement**	**Cohen's Kappa Statistic (95% Confidence Interval)**
Hospital 1	44.8	0.31 (0.30 - 0.33)
Hospital 2	47.4	0.33 (0.32 - 0.35)
Hospital 3	48.5	0.35 (0.34 - 0.37)
Hospital 4	51.4	0.37 (0.36 - 0.39)
Hospital 5	49.8	0.37 (0.35 - 0.38)
Hospital 6	40.1	0.25 (0.24 - 0.26)
Hospital 7	50.6	0.36 (0.34 - 0.38)
Hospital 8	46.8	0.34 (0.32 - 0.35)

© 2024 Stephens CQ et al. *JAMA Network Open*.

eTable 4. Spearman's correlation coefficients of SDoH indices classifying patients within the same quintile with 2-index comparisons

**Area Deprivation Index and Social Vulnerability Index**			
**Hospital Site**	**N**	**Spearman's Rho**	**p-value**
Hospital 1	4776	0.51	<0.001
Hospital 2	8036	0.65	<0.001
Hospital 3	8498	0.69	<0.001
Hospital 4	5196	0.72	<0.001
Hospital 5	6334	0.68	<0.001
Hospital 6	7484	0.54	<0.001
Hospital 7	5054	0.68	<0.001
Hospital 8	7795	0.66	<0.001
**Area Deprivation Index and Child Opportunity Index**			
**Hospital Site**	**N**	**Spearman's Rho**	**p-value**
Hospital 1	4690	0.62	<0.001
Hospital 2	8084	0.74	<0.001
Hospital 3	8305	0.76	<0.001
Hospital 4	4967	0.75	<0.001
Hospital 5	6334	0.77	<0.001
Hospital 6	7455	0.67	<0.001
Hospital 7	4932	0.70	<0.001
Hospital 8	7678	0.74	<0.001
**Child Opportunity Index and Social Vulnerability Index**			
**Hospital Site**	**N**	**Spearman's Rho**	**p-value**
Hospital 1	4714	0.79	<0.001
Hospital 2	8082	0.76	<0.001
Hospital 3	8378	0.79	<0.001
Hospital 4	4984	0.80	<0.001
Hospital 5	6360	0.76	<0.001
Hospital 6	7572	0.71	<0.001
Hospital 7	5051	0.80	<0.001
Hospital 8	7719	0.75	<0.001

© 2024 Stephens CQ et al. *JAMA Network Open*.

eReferences

1. Grubesic TH. Zip codes and spatial analysis: Problems and prospects. *Socioecon Plann Sci*. 2008;42(2):129-149. doi:10.1016/j.seps.2006.09.001

2. Grubesic TH, Matisziw TC. On the use of ZIP codes and ZIP code tabulation areas (ZCTAs) for the spatial analysis of epidemiological data. *Int J Health Geogr*. 2006;5(1):58. doi:10.1186/1476-072X-5-58

3. United States Census Bureau. Standard Hierarchy of Census Geographic Entities. Accessed December 15, 2023. https://www2.census.gov/geo/pdfs/reference/geodiagram.pdf

4. Chacon MA, Cook CA, Flynn-O'Brien K, Zagory JA, Choi PM, Wilson NA. Assessing the Impact of Neighborhood and Built Environment on Pediatric Perioperative Care: A Systematic Review of the Literature. *J Pediatr Surg*. 2024;59(7):1378-1387. doi:10.1016/j.jpedsurg.2024.03.033

5. US Department of Commerce, Economics and Statistics Administration, Bureau of the Census. *Geographic Areas Reference Manual*.; 1994. https://www2.census.gov/geo/pdfs/reference/GARM/

6. Goldberg DW. *A Geocoding Best Practices Guide*. North American Association of Central Cancer Registries; 2008. Accessed December 5, 2023. https://www.naaccr.org/wp-content/uploads/2016/11/Geocoding_Best_Practices.pdf

7. Goldstein ND, Auchincloss AH, Lee BK. A No-Cost Geocoding Strategy Using R. *Epidemiology*. 2014;25(2):311. doi:10.1097/EDE.0000000000000052

8. Krieger CT Jarvis T Chen, Enjoli Hall, Dena Javadi, Justin Morgan, Tamara Rushovich, Sudipta Saha, Pamela D Waterman, Nancy. *Public Health Disparities Geocoding Project 2.0 Training Manual*. Accessed December 5, 2023. https://phdgp.github.io/PHDGP2.0/index.html

9. Singh GK. Area Deprivation and Widening Inequalities in US Mortality, 1969–1998. *Am J Public Health*. 2003;93(7):1137-1143. doi:10.2105/ajph.93.7.1137

10. Kind AJH, Buckingham WR. Making Neighborhood-Disadvantage Metrics Accessible - The Neighborhood Atlas. *N Engl J Med*. 2018;378(26):2456-2458. doi:10.1056/NEJMp1802313

11. Breslau J. Landscape of Area-Level Deprivation Measures and Other Approaches to Account for Social Risk and Social Determinants of Health in Health Care Payments. ASPE. September 26, 2022. Accessed December 10, 2023. https://aspe.hhs.gov/reports/area-level-measures-account-sdoh

12. Acevedo-Garcia D, McArdle N, Hardy EF, et al. The child opportunity index: improving collaboration between community development and public health. *Health Aff Proj Hope*. 2014;33(11):1948-1957. doi:10.1377/hlthaff.2014.0679

13. Acevedo-Garcia D, Noelke C, McArdle N, et al. Racial And Ethnic Inequities In Children's Neighborhoods: Evidence From The New Child Opportunity Index 2.0. *Health Aff Proj Hope*. 2020;39(10):1693-1701. doi:10.1377/hlthaff.2020.00735

14. Noelke C, McArdle N, Baek M, et al. *Child Opportunity Index 2.0: Technical Documentation.* Institute for Child, Youth and Family Policy; 2020. Accessed August 28, 2023. https://www.diversitydatakids.org/sites/default/files/2020-02/ddk_coi2.0_technical_documentation_20200212.pdf

15. CDC/ATSDR Social Vulnerability Index (SVI). July 12, 2023. Accessed December 10, 2023. https://www.atsdr.cdc.gov/placeandhealth/svi/index.html

© 2024 Stephens CQ et al. *JAMA Network Open*.

16. Centers for Disease Control and Prevention (CDC). *Planning for an Emergency: Strategies for Identifying and Engaging At-Risk Groups. A Guidance Document for Emergency Managers: First Edition*. CDC; 2015. https://www.cdc.gov/nceh/hsb/disaster/atriskguidance.pdf

17. Yap A, Laverde R, Thompson A, et al. Social vulnerability index (SVI) and poor postoperative outcomes in children undergoing surgery in California. *Am J Surg*. 2023;225(1):122-128. doi:10.1016/j.amjsurg.2022.09.030

© 2024 Stephens CQ et al. *JAMA Network Open*.
