# Supplementary material for: Comparative Analysis of Indices for Social Determinants of Health in Pediatric Surgical Populations
Source: JAMA Netw Open. 2024 Dec 10;7(12):e2449672. doi: 10.1001/jamanetworkopen.2024.49672 (PMC11632545; doi:10.1001/jamanetworkopen.2024.49672)
Supplement: Supplement 2. — Data Sharing Statement [file jamanetwopen-e2449672-s002.pdf]

Data Sharing Statement

Stephens. Comparative Analysis of Indices for Social Determinants of Health in Pediatric Surgical Populations. *JAMA Netw Open*. Published December 10, 2024.
doi:10.1001/jamanetworkopen.2024.49672

Data
Data available: No

Additional Information
Explanation for why data not available: The investigators are unable to share the data utilized in this study due to the data sensitivity and inclusion of personal health information.
